# Cloning, Expression, and Characterization of a Highly Stable Heparinase I from *Bacteroides xylanisolvens*

**DOI:** 10.3390/polym15071776

**Published:** 2023-04-03

**Authors:** Jia-Lu Pei, Wei Wei, Ding-Ran Wang, Cai-Yun Liu, Hua-Ping Zhou, Chen-Lu Xu, Ye-Wang Zhang

**Affiliations:** 1 School of Pharmacy, Jiangsu University, Zhenjiang 212013, China; 2 Zhongshiduqing Biotechnology Co., Ltd., Heze 274100, China

**Keywords:** Heparinase I, thermostability, expression, characterization, molecular docking

## Abstract

Heparinase I (Hep I), which specifically degrades heparin to oligosaccharide or unsaturated disaccharide, has an important role in the production of low molecular weight heparin (LMWH). However, low productivity and stability of heparinase I hinders its applications. Here, a novel heparinase I (BxHep-I) was cloned from *Bacteroides xylanisolvens* and overexpressed in soluble form in *Escherichia coli*. The expression conditions of BxHep-I were optimized for an activity of 7144 U/L. BxHep-I had a specific activity of 57.6 U/mg at the optimal temperature and pH of 30 °C and pH 7.5, with the *K_m_* and *V_max_* of 0.79 mg/mL and 124.58 U/mg, respectively. BxHep-I catalytic activity could be enhanced by Ca^2+^ and Mg^2+^, while strongly inhibited by Zn^2+^ and Co^2+^. Purified BxHep-I displayed an outstanding thermostability with half-lives of 597 and 158 min at 30 and 37 °C, respectively, which are the highest half-lives ever reported for heparinases I. After storage at 4 °C for one week, BxHep-I retained 73% of its initial activity. Molecular docking revealed that the amino acids Asn25, Gln27, Arg88, Lys116, His156, Arg161, Gln228, Tyr356, Lys358, and Tyr362 form 13 hydrogen bonds with the substrate heparin disaccharides in the substrate binding domain and are mainly involved in the substrate binding of BxHep-I. These results suggest that the BxHep-I with high stability could be a candidate catalyst for the industrial production of LMWH.

## 1. Introduction

Heparinases, also known as heparin lyases, are a large group of polysaccharides lyases. By cleaving the specific 1→4 linkages between hexosamine and uronic acids via the β-elimination mechanism, heparinases specifically depolymerize heparin and heparan sulfate to form a series of disaccharide/oligosaccharide products containing 4,5-unsaturated UA (DUA) moieties at the non-reducing ends [1,2,3]. Heparinases are categorized as heparinase I (EC 4.2.2.7), heparinase II, and heparinase III depending on the specificity of the substrate. Heparinase I is highly selective for cleaving highly sulfated regions and heparinase III exhibits high specificity on cleaving undersulfated regions, while heparinase II shows a wide range of substrate specificity [4,5]. Since heparinase I specifically catalyzes the most abundant sulfated disaccharide units (75–95%) in heparin, it is the most active enzyme for heparin compared to heparinase II and III [6,7,8]. Thus, it has a wide range of applications, including the detection and removal of heparin contaminants [9], the sequence analysis and structural confirmation of heparins [10,11], as well as the removal of heparin from patients during extracorporeal therapy [12]. It is also an important catalyst to manufacture LMWH (4000–6000 Da), which is derived from common heparin (10–40 kDa) through chemical or enzymatic depolymerization. As an anticoagulant drug, LMWH offers higher specific activity, higher bioavailability, longer duration of action, and fewer side effects compared with the original heparins [13,14,15]. Compared with the chemical routes, enzymatic production of LMWH using heparinase I is more efficient, environmentally friendly and controllable [16]. To meet the requirements of the industrial applications, heparinases I with high activity and stability have gained increasing attentions for the sharp increasing demand of LMWH.

Heparinase I was first discovered in *Flavobacterium heparinum* [1], while it has been found to be produced by other microorganisms including several *Bacteroides* [17,18,19,20]. Production of native heparinase with the original bacteria is hampered by several obstacles, including variability in production [20], costly heparin-based inducers [21], low productivity, and low purity of the product [19,22]. The expression of heparinase I heterologously could be used as a route to overcome these drawbacks. In *Escherichia coli*, heparinases I from *F. heparinum* and *B. stercoris* have been expressed. However, they are highly susceptible to the formation of inclusion bodies which are insoluble aggregates of misfolded protein, leading to difficult purification and low recovery activity [19,23]. To solve these drawbacks, a fusion of the enzyme with maltose-binding protein (MBP-HepA) could express around 90% the enzyme as soluble protein in *E. coli*. Nevertheless, the activity was too low which is probably because the spatial structure of the enzyme is affected by the fusion tag [24]. Furthermore, *Pichia pastoris* GS115 was also used as the expression host, and the recombinant heparinase I activity was determined to be 398.5 U/L [25] which is too low to match industrial demand. It is known that the expression of the enzymes is affected by their primary structures, so screening novel enzymes with high activity and could be expressed easily as soluble protein is interesting work for researchers.

In another aspect, the poor thermostability and storage stability of heparinase I has also limited industrial applications. For example, native heparinase I from *P. heparinus* lost 40% of its initial activity upon storage at 4 °C for one day, exhibiting low storage stability [2]. The fused heparinase I from *P. heparinus* with maltose binding protein (MBP-HepI) was observed to have a short half-life of only 130 min at 30 °C [26]. High thermostability is favorable for industrial production because it will save the cost of enzymes. From this aspect, the heparinase I with high thermostability will benefit industrial applications.

Herein, a novel heparinase I from *B. xylanisolven* (BxHep-I) exhibiting superior stability was cloned and expressed in *E. coli*. The expression conditions of the BxHep-I were optimized to improve its productivity. Biochemical characterization of purified BxHep-I was carried out to reveal the enzyme was highly stable and with acceptable activity. Furthermore, comparative homology modeling was used to construct the spatial structure of BxHep-I. Molecular docking with heparin was performed to analyze the interaction of enzymes with the substrate.

## 2. Materials and Methods

### 2.1. Chemicals and Reagents

His-tag protein purification kits were purchased from Qianchun Biotechnology Co., (Zhejiang, China). Competent cells (*E. coli* DH5α and BL21) were obtained from Qingke Biotechnology Co., (Beijing, China). The strain *B. xylanisolven* was bought from Guangdong Microbiology Culture Center (Guangzhou, China). Other chemicals were of analytic or biological grade and supplied by Sinopharm (Shanghai, China). Sodium heparin was provided by Shenzhen Glyca Biotechnology Co. Ltd. (Shenzhen, China).

### 2.2. Cloning of BxHep-I Gene from B. xylanisolvens

The *B. xylanisolvens* heparinase I (BxHep-I) gene was amplified from the genome DNA of *B. xylanisolvens* by polymerization chain reaction using the following primers (forward and reverse primers). (F): 5′-CGGGATCCCGACCGTAAATGGCTGACTG-3′; (R): 5′-CCTTCGAATTCCGCCTCTTTGCCATAGA-3′; (Bam HI and Hind III restriction sites are underlined). The amplified PCR product of the BxHep-I gene was purified by agarose gel electrophoresis and ligated into the pET28a expression vector to construct a recombinant plasmid. The plasmid, pET28a- BxHep-I, was transferred into competent *E. coli* BL21(DE3). Positive stains were screened on the LB agar plate containing 50 μg/mL of kanamycin sulfate.

### 2.3. Expression and Purification of BxHep-I

The *E. coli* containing pET28a-BxHep-I was cultured in the 50 mL LB medium with kanamycin sulfate (50 μg/mL) at 37 °C until the OD_600_ value reached 0.6–0.8, then the recombinant *E. coli* was induced with 0.1 mM IPTG at 25 °C for 7 h. The cultivated cells were extracted by centrifugation at 5000× *g* for 10 min after IPTG induction. Then, the cells were washed twice and resuspended in 5 mL100 mM lysis buffer (Tris-HCl). Additionally, put the cells in the ice bath and disrupted the cells with ultrasonication. The supernatant of the sonicated mixture was collected by removing the cell debris with centrifugation at a speed of 8000× *g* for 15 min at 4 °C. The recombinant BxHep-I was then purified with affinity chromatography using a Ni-NTA column. The Bradford method was used to measure the soluble protein concentration [27]. The expression and purity of BxHep-I were evaluated with 12% SDS-PAGE.

### 2.4. Enzyme Assay

The activity of BxHep-I was estimated by measuring the unsaturated double bonds between C4 and C5 at the uronic acid ring (maximum absorption at 232 nm) in the enzymatic reaction product [24]. Briefly, the reaction solution contained 100 μL of 20 g/L sodium heparin solution, 5 μL of BxHep-I, and 895 μL of 100 mM Tris-HCl buffer which was held at 30 °C for 1 min reaction. The amount of degraded substrate was measured at 232 nm using a UV spectrophotometer and the enzyme activity was estimated from the change in absorbance with a molar extinction coefficient of 3800 L/mol·cm. The quantity of enzyme required to produce 1 μmoL of unsaturated oligosaccharides per minute was considered one unit of the specific activity of BxHep-I.

### 2.5. Optimization of BxHep-I Expression

To obtain a high yield of BxHep-I, the expression conditions were optimized. Firstly, the influence of IPTG concentration on the BxHep-I expression was evaluated over a concentration range of 0.05 to 1 mM. To determine the appropriate temperature for BxHep-I expression, the following temperatures were selected to incubate the bacteria: 15, 20, 25, 30, 35, and 37 °C. To investigate the optimum incubation time for BxHep-I expression, the crude enzyme was collected after the fermentation medium had been incubated for the specified amount of time (3–9 h). Afterward, assessment of BxHep-I yields by measuring the crude enzyme activity of the collected supernatants.

### 2.6. Biochemical Characterization of BxHep-I

#### 2.6.1. Effects of Temperature on the Activity and Stability of BxHep-I

The effect of temperature on BxHep-I was evaluated in a temperature range of 15~60 °C. The buffer was incubated at different temperatures for 5 min and then the catalytic activity of BxHep-I on the substrate heparin was measured.

To investigate the thermal stability of BxHep-I, it was placed in Tris-HCl buffer (pH 7.5) at 30 and 37 °C for different intervals. The initial activity of each sample was identified as 100%. The half-life was calculated by measuring its residual activity after incubation. For storage stability, the Tris-HCl buffer (50 mM, pH 7.5) containing 1.0 mg/mL purified BxHep-I was stored at 4 °C and its residual activity was determined.

#### 2.6.2. Effect of pH on the Activity and Stability of BxHep-I

To check the influence of pH on the activity of BxHep-I, the enzymatic reactions were performed under standard conditions in the following buffers with various pH values: citrate buffer for pH 4.5–6.5, Bis-Tris buffer for 6.5–7.0, and Tris-HCL buffer for pH 7.0–9.0. For pH stability, the residual activity of BxHep-I was measured for 6 h incubation at 30 °C in different buffers with various pH values described above. Each experiment was carried out at least three times.

#### 2.6.3. Effects of Metal Ions and EDTA on the Activity of BxHep-I

The effect of metal ions on the enzyme was determined using standard enzyme assay conditions in the presence of 10 mM Ca^2+^, Co^2+^, Mg^2+^, Ni^2+^, Mn^2+^, Zn^2+^, Na^+^, K^+^, and Li^+^ in Tris-HCl buffer. The effect of EDTA on the enzyme activity was also evaluated and parallel reactions without metal ions or EDTA were set as controls.

### 2.7. Enzyme Kinetics

The kinetic parameters were established by measuring the enzyme initial reaction rate under different heparin concentrations ranging from 0.01 to 2.0 mg/mL under optimal reaction conditions. The *V_max_* and *K_m_* of BxHep-I were determined using non-linear regression by fitting the Michaelis-Menten equation.

### 2.8. Homology Modeling and Substrate Docking of BxHep-I

In order to find homologous templates, the BxHep-I sequence was evaluated using BLAST, multiple sequence alignments were carried out using Clustal-W and the results were presented using ESPript 3.0. The crystal structure of heparinase I from *Bacteroides thetaiotaomicron* (PDB 3ikw, resolution 1.3 Å) was used as the template. The tertiary structure of BxHep-I was predicted by Swiss-model and evaluated in SAVES.

To determine the catalytic residues of BxHep-I, molecular docking was performed. Ligand heparin was downloaded from PubChem. The ligand and receptor models were then prepared using AutoDockTool. Docking of ligands to BxHep-I was performed using AutoDock vina, and the results were visualized using PyMOL.

## 3. Results and Discussion

### 3.1. Sequence Analysis of BxHep-I

The whole genome encoding heparinase I of *B. xylanisolvens* was 1145 bp and encoded 381 amino acids. A putative signal peptide containing 22 amino acids at the N-terminus was detected by SignalP (version 5.0) [28]. According to ExPASy calculation https://www.expasy.org/ (accessed on 1 February 2023), the encoded protein comprised 381 amino acids had a molecular mass of 43.05 kDa. The calculation indicated that the mature protein, excluding the predicted signal peptide, had a theoretical molecular mass of 40.69 kDa and an isoelectric point (pI) of 8.86.

**Figure 1:**
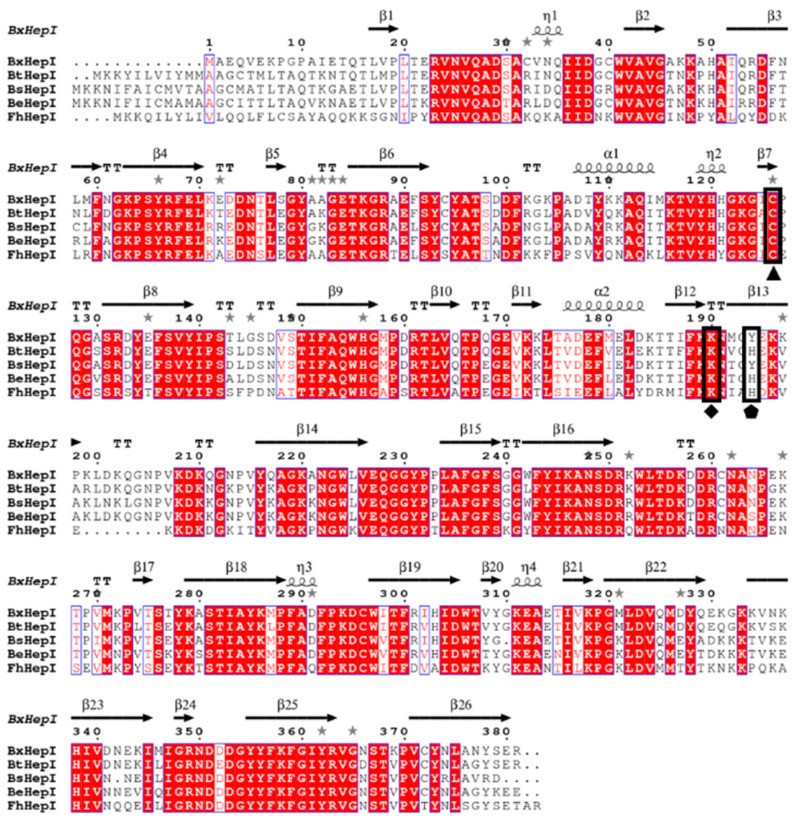
Multiple sequence alignment of heparinases I from *B. xylanisolvens* (BxHep-I), *B. thetaiotaomicron* (BtHep-I), *B. stercoris* (BsHep-I), *B. eggerthii* (BeHep-I), and *F. heparinum* (FhHep-I).

A multiple sequence alignment of heparinases I from *B. xylanisolvens* (BxHep-I), *B. thetaiotaomicron* (BtHep-I), *B. stercoris* (BsHep-I), *B. eggerthii* (BeHep-I), and *F. heparinum* (FhHep-I) is presented in Figure 1. The BxHep-I sequence exhibited 60.5%, 80.9%, 78.2%, and 76.9% identifies with FhHep-I, BtHep-I, BsHep-I and BeHep-I, respectively. As illustrated in Figure 1, His-203 (labeled with a pentagon), which has been identified as a catalytic residue initiating the β-elimination reaction in FhHep-I [29], is replaced with Tyr-203, a nucleophilic residue, in BxHep-I. In addition, Cys-135 is thought to be a crucial nucleophilic amino acid involved in the cleavage of substrates in FhHep-I [30]. Lys199 is considered a calcium-binding site that was essential for the enzyme activity of FhHep-I [31]. These two regions are highly conserved in BxHep-I, which might contribute to the catalytic activity and structure (labeled with a triangle and diamond, respectively).

### 3.2. Cloning, Expression, and Purification of BxHep-I

**Figure 2:**
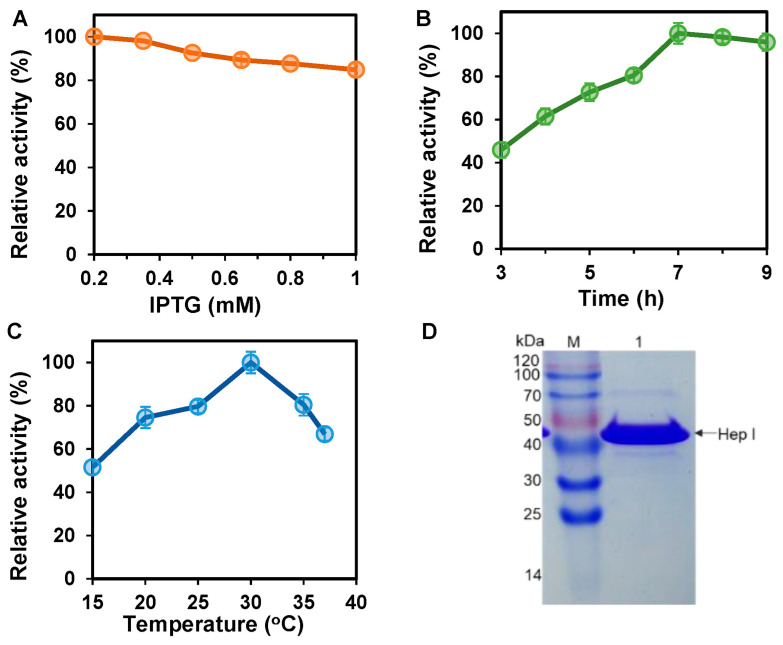
Expression and purification of BxHep-I. (**A**) Effect of IPTG concentration, (**B**) inducing time, and (**C**) temperature on the BxHep-I activity. (**D**) The SDS-PAGE image of the purified BxHep-I (M: protein molecular weight marker; lane 1: the purified BxHep-I).

The coding sequence of BxHep-I without the signal peptide was amplified and subcloned into the pET-28a(+) expression vector. The recombinant protein with an N-terminal His-tag was expressed in *E. coli*. BxHep-I was clearly presented as a soluble protein when the recombinant *E. coli* were induced with IPTG. The effects of temperature, IPTG concentration and inducing time on enzyme expression were analyzed. As demonstrated in Figure 2A, the highest enzyme activity of BxHep-I recording 3772 U/L was achieved at the 0.2 mM of IPTG. However, the activity of BxHep-I decreased slightly with the increase in the IPTG concentration. This might be explained by the cytotoxicity of IPTG [32], which indicates that high concentrations of IPTG in the medium might prevent bacterial reproduction and enzyme production.

The optimal inducing time for BxHep-I expression was investigated by determining the crude enzyme activity at the indicated time points after induction with IPTG. Following a 7 h incubation period, the highest volumetric activity of BxHep-I was obtained, producing 4715 U/L, as shown in Figure 2B. After 7 h induction, the expression level of BxHep-I gradually reduced. This may be due to the overexpression of proteins and polypeptides in bacterial growth, which may provoke protein aggregation and inclusion body formation [33]. Moreover, insufficient nutrients in the medium may lead to the restrain of enzyme generation.

The inducing temperature has a strong effect on the expression of BxHep-I. As shown in Figure 2C, the maximum activity of BxHep-I was obtained at 30 °C, recording 7144 U/L With the induction temperature increased from 25 to 37 °C, the BxHep-I activity significantly declined. This might be attributed to the rapid expression of the recombinant protein at high temperatures suitable for *E. coli* growth, which resulted in protein misfolding [34,35]. In summary, the optimal expression of BxHep-I was observed at the following conditions: 0.2 mM of IPTG, temperature 30 °C, and the induction time was 7 h, the total enzyme activity was 7144 U/L.

Chelated nickel ions affinity column chromatography was used to purify the recombinant heparinase I, an in-frame fusion protein with His-tag, to apparent homogeneity. According to Figure 2D, the molecular mass of heparinase I was calculated as 43 kDa by measuring the position of the pure BxHep-I to the protein standard marker. The molecular weight is consistent with the estimated molecular weight of heparinase I. Purified BxHep-I had a specific activity of 57.6 U/mg using heparin as the substrate.

### 3.3. Biochemical Characterization of BxHep-I

**Figure 3:**
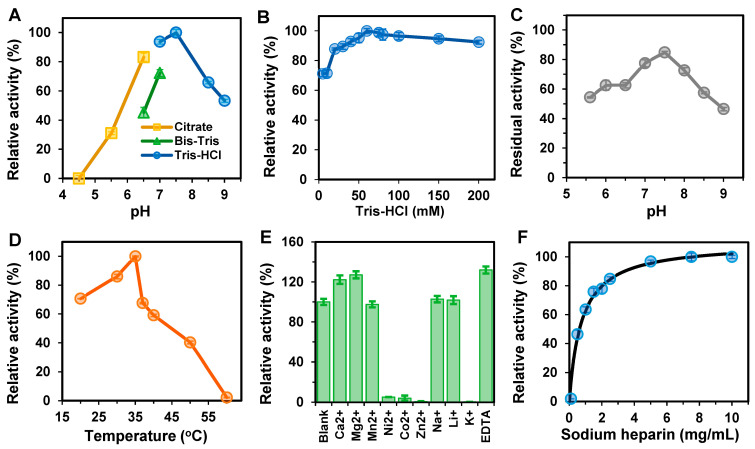
Characterization of the purified BxHep-I. Effects of (**A**) pH, (**B**) Tris-HCl concentration, (**D**) Temperature, (**E**) Metal ions and EDTA, and (**F**) Substrate concentration on the activity of BxHep-I. (**C**) The effect of pH on the stability of BxHep-I.

Figure 3A shows how pH impacted the activity of pure BxHep-I. The optimal pH for BxHep-I was 7.5. Additionally, the enzyme showed high enzymatic activity in a wide range of pH, with more than 80% of its maximal activity in the pH range of 6.5–8.0. The residual activity was found to be 53% at a pH of 9.0. However, BxHep-I was almost inactive in citrate buffer at pH 4.5, and only 31% of enzyme activity was retained at pH 5.5. This indicates that BxHep-I has excellent alkali resistance. Our findings are consistent with some other research, where BtHep-I and FhHep-I exhibited high enzymatic activity in the pH range of 6.5–7.5 [36,37], and BeHep-I and BcHep-I showed the same optimum pH [17,18]. Additionally, BxHep-I activity was measured at Tris-HCl concentrations ranging from 5 to 200 mM. Figure 3B showed that the optimal Tris-HCl concentration for BxHep-I was 60 mM. The relative activity improved from 71% to the highest with an increase in concentration from 5 to 60 mM. More than 90% of the optimum activity was maintained when the Tris-HCl concentration was raised to 200 mM. Figure 3C presents the pH stability of BxHep-I. After 6h incubation at 30 °C, the enzyme retained most of its catalytic ability (more than 77.69% residual activity) with the pH range 7.0 to 8.0. In a wide pH range from 5.0 to 8.5, BxHep-I with more than 54.50% residual activity exhibited good pH stability. At pH 7.5, BxHep-I showed maximal activity and pH stability indicating that the enzyme was better tolerated in a weakly alkaline environment.

Figure 3D demonstrates that BxHep-I was active over a wide temperature range from 20 to 60 °C, with the highest activity occurring at 35 °C and measuring 52 U/mg. The enzyme activity steadily increased from 71% to 100% with the temperature changed from 20 to 35 °C. Additionally, it declined rapidly when the temperature exceeded 35 °C and was almost lost at 60 °C, which could be related to the significant damage to the tertiary structure and configuration of protein caused by high temperatures, which further inhibited enzyme activity. According to the previous reports, the optimum temperatures of FhHep-I and BeHep-I were 30 °C [18,26], and BsHep-I was found to have the maximal activity at 37 °C [19].

To determine the effects of different metal ions and EDTA on the enzyme activity, the BxHep-I was tested under 10 mM metal ions or EDTA (Figure 3E). Ca^2+^, Mg^2+^, and EDTA could significantly increase the activity of BxHep-I, reaching by 115%, 127%, and 132% relative activity compared with the control, respectively. However, Zn^2+^, Ni^2+^, K^+^ and Co^2+^ strongly inhibited the activity of BxHep-I. Specifically, Co^2+^ and Ni^2+^ reduced the enzyme activity by 94.89% and 95.98%, respectively. Additionally, the enzyme could be inactivated by Zn^2+^ and K^+^. It is apparent that Li^+^ and Na^+^ have no discernible effect on the BxHep-I activity. It has been reported that heparinase I activity was enhanced by Ca^2+^ [17,18,19,38] because Ca^2+^ could neutralize the carboxyl moiety of the IduUA at the cleavage site, thus specific binding of Ca^2+^ to two major calcium-binding sites on heparinase I could participate in the direct catalysis and the exolytic processive [39,40]. In the present work, Mg^2+^ also can improve the specific activity which could broaden the applications in the case that Ca^2+^ is not allowed in the reactions.

The kinetic parameters of BxHep-I with heparin as substrate were determined under optimized reaction conditions (Figure 3F). The results computed by the Michaelis-Menten equation and double-reciprocal plot demonstrated that BxHep-I has a *K_m_* of 0.79 mg/mL and a *V_max_* of 124.58 U/mg. As shown in Table 1, compared to the previous reports, the *V_max_* of BxHep-I was 2.4-fold of BsHep-I, but only 56% of FhHep-I, 19% of BeHep-I, and 16% of BcHep-I, indicating that BxHep-I has a lower catalytic efficiency. The *K_m_* of BxHep-I was 4.6-fold of BcHep-I indicating that the enzyme has a lower affinity for the substrate heparin. Those were also evidenced by the fact that the specific activity of BxHep-I was similar to that of MBP-fused FhHep-I and BsHep-I, but lower than other heparinases I.

### 3.4. Thermostability Analysis of BxHep-I

It is extremely critical to assess the thermostability of enzymes because high temperatures may seriously damage their intramolecular forces, which maintain the tertiary structure and configuration of the enzyme. The enzyme’s affinity for the substrate may be inhibited by changes in enzyme conformation that result in a decrease in the catalytic activity of the enzyme.

**Figure 4:**
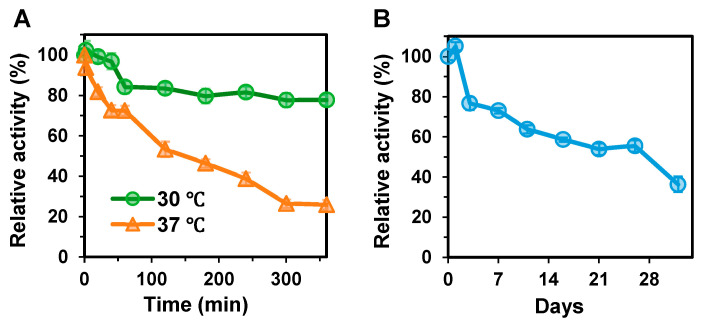
The stability of the recombinant heparinase I. (**A**) The thermostability of BxHep-I at 30 and 37 °C and (**B**) the storage stability at 4 °C.

The thermostability of BxHep-I was determined at 30 and 37 °C, separately, as illustrated in Figure 4A. After 40 min of incubation, BxHep-I retained nearly all its initial activity at 30 °C, then steadily decreased and maintained 78% of its maximum activity after 6 h. The results estimated through linear fitting showed that the half-life of BxHep-I at 30 °C was 597 min, whereas the half-life of the recombinant FhHep-I was only 10 min [41]. At 37 °C, the enzyme maintained over 70% of its maximum activity after 1 h of incubation and after 3 h, 46% of initial activity was determined. BxHep-I has a half-life of 158 min at 37 °C, while FhHep-I has only 3 min under the same conditions [41].

Figure 4B illustrates the storage stability of BxHep-I at 4 °C. The enzyme, with a half-time of 3 weeks, retained 77% and 56% of its initial activity after 3 and 26 days of storage at 4 °C, respectively.

To our knowledge, BxHep-I possesses the best thermal stability ever reported. Table 1 displays the stability of heparinases I from different organisms where the half-life of BxHep-I at 30 °C is 59.7-fold of MBP-fused FhHep-I, 4.59-fold of FhHep-I mutant, 1.7-fold of BeHep-I and 1.99-fold of BxHep-I. The half-life at 37 °C of BxHep-I is also distinguished, which is 5.27-fold of BsHep-I, 2.63-fold of BeHep-I, and 2.68-fold of BcHep-I. The storage stability at 4 °C is similar to that of FhHep-I mutant and BeHep-I, but substantially better when compared to other heparinases I. For example, after 5 days of storage at 4 °C, FhHep-I only retained 20% of its initial activity [2,24]. These results exhibited that BxHep-I has practical applications in both clinical trials and industrial production.

Disruption of the tertiary structure of the protein is an essential factor in protein inactivation [42]. In addition, the heparin binding sites were found to include a calcium coordination consensus motif and two Cardin-Weintraub heparin binding consensus sequences, which may aid the structural integrity of the enzyme [43,44]. Therefore, Ca^2+^ has a potential role in promoting heparinase I stability by maintaining the protein tertiary structure.

It was also proposed that the application of Ca^2+^ at an optimal concentration might prevent the unfolding of heparinase I and improve its stability [26,45]. For instance, after 30 min of incubation at 30 °C, MBP-Hep-residual activity was enhanced 1.5 times to the control when 1 mM Ca^2+^ was added [26]. Therefore, it is noted that the presence of Ca^2+^ tends to enhance the thermostability and activity of BxHep-I which include calcium-binding sites.

**Table 1:** Comparison of heparinases I from various species.

Organisms	Heparinase I	Specific Activity (U/mg)	Kinetic Parameters		t_1/2_ (min)		Residual Activity (%) *	Reference
			*K_m_* (mg/mL)	*V_max_* (U/mg)	30 °C	37 °C		
*F. heparinum*	Native	90.33	17.8	219.48	-	-	<30	[45]
*F. heparinum*	Recombinant MBP-fused	149	-	-	10	-	11.3	[26]
*F. heparinum*	Recombinant MBP-fused mutant	56.31	-	-	130	-	85.1	[46]
*B. stercoris*	Recombinant	46.02	0.023	51.81	-	30	-	[19]
*B. eggerthii*	Recombinant	480	3.6	647.93	350	60	89.1	[17]
*B. cellulosilyticus*	Recombinant	738.8	0.17	750.58	300	59	58.6	[18]
*B. xylanisolvens*	Recombinant	57.6	0.79	124.58	597	158	73.1	This work

*: residual activity of Hep-I after 1-week of storage at 4 °C.

### 3.5. Homology Modeling and Substrate Docking

**Figure 5:**
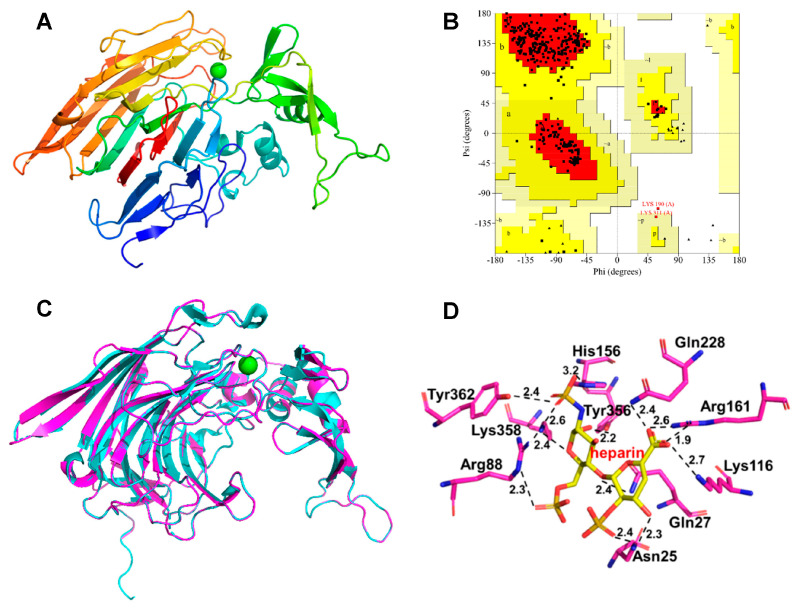
Homology modeling of BxHep-I and molecular docking with heparin. (**A**) a model of BxHep-I, (**B**) Ramachandran plot of BxHep-I, (**C**) The superimposition of the template (BtHep-I, 3ikw, blue) and BxHep-I (red), (**D**) molecular docking of BxHep-I with heparin).

The structure of BtHep-I (PDB: 3IKW, 80.9% agreement with the query sequence) was found to be the most appropriate homologous template according to BLAST results. Swiss-model was used to construct and SAVES online server was used to evaluate the three-dimensional structure of BxHep-I (Figure 5A). The Ramachandran plot obtained from Procheck software depicted 99.7% of the residues in generously allowed regions and only 0.3% in disallowed regions, which shows that most residues follow the phi/psi angle distribution and that the model is reliable and of high quality (Figure 5B). According to the Verify 3D compatibility of the consistency between primary sequences of proteins and the simulated tertiary structure, 96.2% of the residues obtained a 3D-1D score of ≥0.2. A statistical analysis of the non-bonded interactions between various types of atoms was used to produce the ERRAT plot, which resulted in a quality factor for the structural model of 96.64. Structural alignment diagrams of BxHep-I and BtHep-I demonstrate that they are structurally similar, and both have calcium-binding domains (Figure 5C). These results further implied that the predicted BxHep-I structure can be used for molecular docking.

The interactions between heparin substrates and BxHep-I were examined using macromolecular docking (Figure 5D). According to the previous reports, the heparin-binding domain creates a basic environment favorable to the cleavage process and offers the charge complementarity required for the specific binding of heparin [44]. The docking model revealed that the active sites Asn25, Gln27, Arg88, Lys116, His156, Arg161, Gln228, Tyr356, Lys358, and Tyr362 in the active center of BxHep-I were highly conserved. These residues are crucial for both the substrate binding and catalytic hydrolysis of heparin.

The binding between enzymes and the substrate was analyzed with the number of formed hydrogen bonds. Figure 5D depicted that there were 13 hydrogen bonds formed with heparin. Asn25 interacts with the heparin atoms O-25 and O-33 to form hydrogen bonds of length 2.3 Å and 2.4 Å, respectively. Gln27, Arg88, and His156 interact with O-3, O-23, and O-28 atoms of heparin with hydrogen bonds of 2.4 Å, 2.3 Å, and 3.2 Å, respectively. Between Lys116 and the O-35 on the carboxyl group of heparin, a hydrogen bond of 2.7 Å was created. Additionally, Gln228 binds to the O-35 atom with a 2.4 Å hydrogen bond whereas Arg 161 connects with the O-34 atoms of heparin through two hydrogen bonds (1.9 Å and 2.6 Å, respectively). With two hydrogen bonds of 2.2 Å and 2.4 Å, Tyr 356 and Try358 bind the O-4 and O-30 of heparin. Try363 forms a 2.4 Å hydrogen bond link with the O-23 on the sulphate group of heparin. Compared with other heparinases I, BxHep-I forms more hydrogen bonds with the substrate which contributes to the stability of the protein-ligand complex. These in silico work suggest that further work on this enzyme could focus on these residues involved in the binding substrate or catalysis.

The conformational entropy of BxHep-I was compared with the other four heparinases I. In comparison to other heparinases, BxHep-I had a lower conformational entropy value of 721 J/K (768 J/K of FhHep-I, 774 J/K of BtHep-I, 756 J/K of BeHep-I and 782 J/K of BsHep-I, respectively) indicating that its conformation was more stable. This proved that BxHep-I had better thermal stability than other enzymes.

In addition, the substrate binding pocket of BxHep-I showed more sterically bulky amino acid residues Arg88, His156, Tyr356 and Tyr362, which could form the larger steric hindrance and block the maneuver and binding of heparin to the catalytic site. As a result, a significant decrease in both catalytic velocity and affinity was shown when sterically bulky arginine, histidine, and tyrosine were in the presence of the enzyme, which is in agreement with the experimental results with the higher *K_m_* value and lower specific activity.

## 4. Conclusions

A novel heparinase I was cloned from *B. xylanisolvens* and overexpressed in soluble form. The purified BxHep-I exhibited a *V_max_* of 57.6 U/mg under optimal reaction conditions (pH 7.5 and 35 °C). The t_1/2_ of BxHep-I was 597 min and 158 min at 30 and 37 °C, respectively, which is the best thermal stability ever reported for heparinases I. In addition, BxHep-I I also exhibits excellent storage stability. Compared with other heparinase I, the high stability of the BxHep-I demonstrates its potential for industrial production of LMWH.

## Data Availability

The data presented in this work are available on request from the corresponding author.
